# Dreaming as a story-telling instinct

**DOI:** 10.3389/fpsyg.2013.00159

**Published:** 2013-04-02

**Authors:** Edward F. Pace-Schott

**Affiliations:** Department of Psychiatry, Harvard Medical School and Massachusetts General HospitalCharlestown, MA, USA

Dreams create new stories out of nothing. Although dreams contain themes, concerns, dream figures, objects, etc. that correspond closely to waking life, these are only story elements. The story itself weaves these mnemonic items together in a manner far more novel than a simple assemblage or collage, producing an experience having a life-like timeframe and life-like (if often bizarre and impossible) causality (Pace-Schott, 2007; Hobson, 2009). It is as if one is immersed in another “reality” entirely of one's own non-volitional, making (see Rechtschaffen, 1978). Given this phenomenology, it's not difficult to see why some indigenous animist societies believe dreams represent a separate world parallel with waking life (Nielsen, 1991). But neuroscience offers some other explanations.

Recent speculations have focused on the brain's “default network” as a possible neural substrate of dreaming (Pace-Schott, 2007, 2011a,b; Nir and Tononi, 2010; Wamsley and Stickgold, 2010; Domhoff, 2011). The default network consists of regions that, in the absence of exteroceptive attention or narrowly focused mental effort, support self-directed concerns, immersion in one's inner life (e.g., daydreaming) or imagining the inner life of others (Theory of Mind) (Buckner et al., 2008; Andrews-Hanna, 2012; Buckner, 2012). Most importantly for the current topic, the default network also simulates future scenarios and re-creates past ones drawing upon material in episodic, autobiographical, and semantic memory (Schacter et al., 2007; Schacter, 2012). Here I will suggest that such constructive activities of the brain represent a “hard-wired” tendency to represent reality in the form of narrative—a “story-telling” instinct or module.

The default network was originally identified using positron emission tomography (PET) as those regions showing task-induced deactivation (Gusnard et al., 2001; Raichle et al., 2001). Subsequently, it was discovered that temporal synchrony of low frequency (0.01–0.1 Hz) spontaneous fluctuations of the blood-oxygen dependent (BOLD) signal of fMRI identifies both anatomical and functional connectivity among regions of the default network (Fox and Raichle, 2007; Greicius et al., 2008). This network consists of (1) medial parietal areas: posterior cingulate (pCC) and retrosplenial (Rsp) cortices; (2) posterior-lateral areas: inferior parietal lobule (IPL), temporo-parietal junction (TPJ), lateral temporal cortex (LTC), temporal poles (TP); (3) medial temporal regions: hippocampal formation (HF), parahippocampal cortex (PHC); and (4) medial prefrontal areas: ventromedial (vmPFC) and dorsomedial (dmPFC) prefrontal cortices (Buckner et al., 2008; Spreng et al., 2009; Andrews-Hanna, 2012).

Resting state functional connectivity analyses of BOLD oscillations in waking have identified two default-network subsystems each of which fluctuates synchronously with central nodes in the pCC and anterior medial PFC (amPFC) but not with each other (Buckner et al., 2008; Andrews-Hanna et al., 2010; Andrews-Hanna, 2012). The dorsomedial prefrontal subsystem includes the dmPFC, LTC, TPJ, and TP whereas the medial temporal lobe subsystem includes the HF, PHC, Rsp, IPL, and vmPFC. The dorsomedial prefrontal subsystem selectively activates during experimental tasks involving reflection on one's own mental state and that of others as well as other forms of social cognition (Andrews-Hanna et al., 2010; Mar, 2011; Andrews-Hanna, 2012). In contrast, the medial temporal lobe subsystem is selectively activated by retrieval of episodic and autobiographical memories as well as by imagination of future scenarios and concerns (Schacter et al., 2007; Andrews-Hanna et al., 2010; Andrews-Hanna, 2012; Schacter, 2012). The central nodes activate along with most tasks that recruit one or the other subsystem (Andrews-Hanna, 2012).

Synchrony of BOLD fluctuations among components of the default network persists into light (Drummond et al., 2005; Horovitz et al., 2008; Larson-Prior et al., 2009) and Stage 2 (Laufs et al., 2007) NREM sleep. However, in slow-wave sleep (SWS), frontal regions may uncouple from the rest of the default network (Horovitz et al., 2009; Samann et al., 2011, but see Koike et al., 2011). In the one study examining REM, unlike both waking and NREM, there appeared a lack of connectivity between the dorsomedial prefrontal subsystem and the posterior central node of the default network in the pCC (Koike et al., 2011). Koike et al. speculate that this disconnection contributes to the illogic and bizarreness of dream cognition, as has also been suggested for loss of antero-posterior EEG synchrony in the fast, gamma (>30 Hz) frequencies during REM (Corsi-Cabrera et al., 2003, 2008).

Earlier PET and fMRI *activational* studies of sleep also showed distinctly different activity in medial limbic versus lateral association cortex during REM sleep. After sleep onset during NREM, widespread cortical and subcortical areas become less active (Braun et al., 1997; Maquet et al., 1997; Nofzinger et al., 2002; Kaufmann et al., 2006). However, with the onset of REM sleep, midline limbic regions of the frontal cortex and subcortex reactivate to levels equaling and sometimes exceeding those of waking, whereas lateral and posterior-medial cortical areas remain in a NREM-like deactivated state (Maquet et al., 1996, 2005; Braun et al., 1997, 1998; Nofzinger et al., 1997, 2004). This unique pattern of REM activation has been extensively mapped onto dream experiences (Hobson et al., 2000; Hobson and Pace-Schott, 2002; Hobson, 2009; Pace-Schott, 2011a). NREM dreams may arise from more transient activations of similar networks (see Nielsen, 2000). Abnormally intensified dreaming, such as nightmares (Levin and Nielsen, 2007), or loss of dreaming (Solms, 1997) may represent, respectively, a failure to regulate or support operation of this limbic network.

Therefore, based on activational studies, the anterior central node (amPFC) and much of the mediotemporal subsystem (e.g., vmPFC, HF, PHC) of the default network may be similarly active in waking and REM. However, the posterior key node (pCC) and certain components of both the dorsomedial prefrontal (TPJ, LTL) and mediotemporal (Rsp, IPL) subsystems may not become similarly active. The one functional connectivity study of REM also suggests this disconnection between the pCC and dmPFC. Therefore, during REM-sleep dreaming, simulation of life-like scenarios may occur but be incompletely constrained by autobiographical memory (both functions of the mediotemporal subsystem). Similarly, in comparison to resting-state waking, self-referential and social cognitive reasoning subserved by the dorsomedial prefrontal system may be less integrated with autobiographical memory.

But do dreams actually tell stories? Cipolli and Poli (1992) applied a formal, story grammar developed to describe narrative texts (Mandler and Johnson, 1977) to REM-dream reports collected by instrumental laboratory awakenings. Formal elements of dream narratives resembled typical stories, for example, by inclusion of characters, settings and a hierarchical event structure (Cipolli and Poli, 1992). Moreover, these elements remained stable between the nocturnal report and its retrospective morning report suggesting that story-like structure was a feature of the dream experience itself rather than being imposed upon its recall during waking. Cippolli and Poli suggest that higher-order cognitive processes organize dream experience (Cipolli et al., 1998). However, story structure may also be the basic manner in which brain organizes experience. Although obscured by temporal progression of events and of cause and effect in waking, this tendency may become more apparent during the non-volitional process of dreaming and pathological states such as delirium or confabulation (Solms, 1997; Hobson, 1999; Schnider, 2003, 2008; Hirstein, 2005).

Like dreaming, spontaneous behavioral confabulation involves a fictive narrative produced effortlessly, without insight as to its veracity, that is often acted upon by the patient (Schnider, 2003, 2008; Hirstein, 2005; Gilboa et al., 2006; Nahum et al., 2012). Spontaneous confabulation results from lesions of the anterior limbic system including posterior medial orbitofrontal cortex (pmOPFC, part of vmPFC) and its subcortical connections (Schnider, 2003, 2008; Hirstein, 2005; Gilboa et al., 2006). Confabulated memories usually contain real autobiographical events including those from the remote past (Schnider, 2003, 2008; Hirstein, 2005). Such loss of insight has been attributed to disruption of a reality filtering function localized to the vmPFC/pmOPFC (Schnider, 2003, 2008) or to a more general deficit in strategic retrieval and verification of memories (Gilboa et al., 2006). In either case, however, phenomenological similarities exist with dreaming. For example, confabulators create plausible but false explanations for inconsistencies in their stories (Hirstein, 2005), closely resembling “*ad-hoc* explanations” for improbable dream occurrences (Williams et al., 1992). Additionally, “pathological false recognition” (Hirstein, 2005) in confabulation parallels dreamers' assigning an identity to dream characters perceptually dissimilar to their waking counterpart (Kahn et al., 2000).

Therefore, in both confabulation and dreaming, altered functioning of the prefrontal cortex may release from reality-filtering or executive constraint an innate human tendency to create stories that organize past, present, and future reality. Dreaming may represent a potent, naturally occurring form of confabulation in which imaginary events are not only created and believed but are vividly experienced as organized, multimodal hallucinations (Hobson, 1999; Pace-Schott, 2007, 2011a). Of course, hallucinosis differentiates dreaming and confabulation as does the fact that vmPFC/pmOPFC *damage* leads to confabulation whereas its *activation* accompanies dreaming. Nonetheless, in both phenomena, involuntary generation of an organized, fictive narrative entirely lacking insight suggests that they may share neural mechanisms. Possibly, the loss of pre-frontally mediated reality monitoring, due either to pmOPFC damage in confabulation or lateral frontal inactivity during dreaming, may release narrative-production mechanisms from inhibitory restraint.

But what about normal narrative production? Braun et al. (2001) performed PET conjunction analysis to identify modality-independent regions activated by storytelling in both English and American Sign Language. Although activity was shared in widespread medial and lateral cortical areas, medial prefrontal activation could be most directly related to the generic production of narrative discourse apart from the imagery, lexical, and memory processes shared by the two modes of storytelling (Braun et al., 2001). Therefore, portions of this same network may be engaged in volitional storytelling (see Mar, 2004 for review).

However, this putative storytelling module expressed as dreaming or confabulation rarely yields levels of organization equal to volitional narrative. For example, discontinuities in dream plots are sufficiently common that judges are unable to distinguish artificial reports created by splicing together text from different dreamers' reports from intact dreams (Stickgold et al., 1994). Nonetheless, the brain may attempt to impose wake-like temporal causality on any experience, real, or imagined. Dream hallucinosis itself may generate low-level narrative coherence by associative processes in which images evoke related images (see Rittenhouse et al., 1994) that are successively woven together by this putative tendency to organize experience as a story (Pace-Schott, 2005). In dreaming, diminished capacity for working memory, due to lateral prefrontal inactivity, may prevent reflection upon immediately past events leading to an unquestioned, forward progression of the plot as well as frequent narrative divergence. Interestingly, recent studies of lucid dreaming have shown both return of wake-like, gamma-frequency activity in the lateral PFC (Voss et al., 2009) and re-activation of portions of posterior default network along with lateral parietal, prefrontal, and occipito-temporal regions (Dresler et al., 2012).

So what might this storytelling module operating during dreams suggest to individuals wholly unfamiliar with neuroscience and scientific psychology? A keen interest in dreams among some hunter-gatherer societies is exemplified by the practice of dream sharing (Wax, 2004). Dreams are sometimes recounted as experiences from another, parallel existence. For example, The Amazonian Ese Eja believe that, during dreaming, their spirit inhabits a parallel world populated by other spirits, including those of animals (Peluso, 2004). Similarly, the Amazonian Mehinaku believe each individual contains several types of souls distinct from their waking selves, one of which, the “eye soul,” is active during the night experiencing dreams but sleeps during the day (Gregor, 1981). Similarly, among the Andamanese Ongee society, discussion of sensory details from multiple individuals' dreams is used to build consensus as to the probable locations of seasonal food sources (Pandya, 2004). Therefore, recalled dreams provide a ready source of story-like narrative that can acquire cultural significance equal to or exceeding the retelling of waking events.

Basic human storytelling tendencies are widely postulated (e.g., Schechtman, 1996; Nigam, 2012; Stafford, 2012). By providing a template into which any experience, real or imaginary, can be inserted, story-telling may be one way the brain has evolved to efficiently represent and record waking experience. Evolution may have subsequently exploited this capacity for multifold purposes including future simulation. Hobson's (2009) protoconsciousness theory, describes how dreams adaptively support waking consciousness. Similarly, evolution may have exploited dreamed scenarios to rehearse survival strategies (Revonsuo, 2000). In indigenous societies, story-like structure may have facilitated integration of dream phenomena such as parasomnias (e.g., sleep paralysis, see Cheyne, 2003), lucidity, partial awakenings and dream bizarreness into existing belief systems, or even to create new beliefs and legends (see Nielsen, 1991).
